# Rapid analysis of hydrogen cyanide in fresh cassava roots using NIRSand machine learning algorithms: Meeting end user demand for low cyanogenic cassava

**DOI:** 10.1002/tpg2.20403

**Published:** 2023-11-08

**Authors:** Michael Kanaabi, Fatumah B. Namakula, Ephraim Nuwamanya, Ismail S. Kayondo, Nicholas Muhumuza, Enoch Wembabazi, Paula Iragaba, Leah Nandudu, Ann Ritah Nanyonjo, Julius Baguma, Williams Esuma, Alfred Ozimati, Mukasa Settumba, Titus Alicai, Angele Ibanda, Robert S. Kawuki

**Affiliations:** ^1^ School of Agricultural Sciences Makerere University Kampala Uganda; ^2^ National Crops Resources Research Institute (NaCRRI) Kampala Uganda; ^3^ International Institute for Tropical Agriculture (IITA) Ibadan Nigeria; ^4^ Plant Breeding and Genetics section Cornell University Ithaca New York USA

## Abstract

This study focuses on meeting end‐users’ demand for cassava (*Manihot esculenta* Crantz) varieties with low cyanogenic potential (hydrogen cyanide potential [HCN]) by using near‐infrared spectrometry (NIRS). This technology provides a fast, accurate, and reliable way to determine sample constituents with minimal sample preparation. The study aims to evaluate the effectiveness of machine learning (ML) algorithms such as logistic regression (LR), support vector machine (SVM), and partial least squares discriminant analysis (PLS‐DA) in distinguishing between low and high HCN accessions. Low HCN accessions averagely scored 1–5.9, while high HCN accessions scored 6–9 on a 1–9 categorical scale. The researchers used 1164 root samples to test different NIRS prediction models and six spectral pretreatments. The wavelengths 961, 1165, 1403–1505, 1913–1981, and 2491 nm were influential in discrimination of low and high HCN accessions. Using selected wavelengths, LR achieved 100% classification accuracy and PLS‐DA achieved 99% classification accuracy. Using the full spectrum, the best model for discriminating low and high HCN accessions was the PLS‐DA combined with standard normal variate with second derivative, which produced an accuracy of 99.6%. The SVM and LR had moderate classification accuracies of 75% and 74%, respectively. This study demonstrates that NIRS coupled with ML algorithms can be used to identify low and high HCN accessions, which can help cassava breeding programs to select for low HCN accessions.

## Core Ideas

- NIRS combined with machine learning algorithms can discriminate low and high HCN cassava accessions with high classification accuracy.
- The wave lengths 961, 1165, 1403–1505, 1913–1981, and 2491 nm are influential in discrimination of low and high HCN accessions.
- Spectral pre‐treatments can increase classification accuracy of machine learning algorithms such as logistic regression and PLS‐DA using the full spectrum range (400–2500 nm).
- NIRS as an accurate, high throughput tool is key for breeding programs to achieve genetic gains in low HCN cassava breeding.

**Abbreviations**
CG: cyanogenic glucoside
HCN: hydrogen cyanide potential
LR: logistic regression
ML: machine learning
NIRS: near‐infrared spectroscopy
PCA: principal component analysis
SG: Savitzky–Golay
SG.d1,w5: Savitzky–Golay with first derivative, window 5
SG.d2,w5: Savitzky–Golay with second derivative, window 5
SG.SNV: Savitzky–Golay with standard normal variate
SNV: standard normal variate
SNV.d1: standard normal variate with first derivative
SNV.d2: standard normal variate with second derivative
SVM: support vector machine

## 1 INTRODUCTION

Cassava (*Manihot esculenta* Crantz) is a major staple for over 800 million people in sub‐Saharan Africa, Asia, the Pacific, and Latin America (Burns et al., 2010). Cassava roots are the primary product of cassava and are a major carbohydrate source, particularly in regions where drought and/or poor soils limit the cultivation of other crops (Okogbenin et al., 2013). Cassava's tolerance to drought and marginal soils and its perennial nature which allows for piecemeal harvest (Burns et al., 2010; El‐Sharkawy, 2012) make it an important food security crop, especially for resource‐poor communities. Cassava roots may be consumed raw, or processed into several food, feed, and industrial products (Iragaba et al., 2021; Nanyonjo et al., 2021; Nuwamanya & Kawuki, 2010).

The full exploitation of cassava's potential is limited by the cyanogenic glucosides (CGs) in both leaves and roots. Linamarin and lotaustralin are the main forms of CGs (Santana et al., 2002), of which linamarin is the most abundant (Padmaja & Steinkraus, 1995). In the presence of linamarase enzyme, linamarin undergoes hydrolysis, producing hydrogen cyanide (Egan et al., 1998). Based on the hydrogen cyanide potential (HCN), cassava varieties are categorized as *sweet* if they have fresh root HCN content <100 ppm and *bitter* if HCN content >100 ppm (Wheatley et al., 2003). Bitter cassava varieties are unsafe for human consumption unless further processed (FAO/WHO, 2001).

Dietary consumption of high HCN cassava roots can result in acute poisoning. The lethal dose of HCN for humans is 0.5–3.5 mg/kg of body weight (Halstrøm & Møller, 1945) and several deaths have been reported due to dietary HCN poisoning (Akintonwa & Tunwashe, 1992; Alitubeera et al., 2019; Teles, 2002). However, prolonged dietary intake of even low levels of HCN is associated with debilitating nervous system disorders—konzo and tropical ataxic neuropathy (Cliff &Nzwalo, 2011; Nhassico et al., 2008). In Uganda, cassava is mainly consumed by boiling or steaming fresh roots (Iragaba et al., 2021) or by processing it into a flour meal (Nanyonjo et al., 2021). Consumers of boiled cassava prefer non‐bitter varieties characterized by low HCN (Iragaba et al., 2021). Moreover, from a recently conducted food product profile inclusive discussion with end users, low HCN was ranked highly, that is, among top four root quality traits that breeders should select (Kawuki et al., 2020). However, breeding progress for reduced HCN has been slow, partly due to the difficulties in phenotyping for HCN. Commonly used methods to measure HCN (Bradbury & Egan, 1992; Cooke, 1978; Egan et al., 1998; Essers et al., 1993; Fukuda et al., 2010) are slow and laborious, which limits both throughput and, in some cases, accuracy. For example, the commonly deployed picrate method (Egan et al., 1998) can only yield results after 12–16 h. Additionally, it involves extensive sample preparation and use of difficult‐to‐handle chemicals like picric acid, which is explosive when dry. Even when efforts were made to improve accuracy of the method using spectrophotometry (Bradbury et al., 1999), this involves more sample preparation steps, thus increased drudgery.

Therefore, the need for faster and accurate HCN screening methods is vital to respond to end user preferences. The use of near‐infrared spectroscopy (NIRS) to predict essential cassava root quality traits is gaining prominence. Thus, NIRS has been deployed to predict cassava root dry matter content and total carotenoids (Abincha et al., 2021, 2020; Belalcazar et al., 2016; Sánchez et al., 2014), starch (Nkouaya Mbanjo et al., 2022), cassava boiled root cooking time (Namakula et al., 2023), and amylose (Nuwamanya, et al., 2022). This progress is hinged on the fact that NIRS offers fast, simultaneous, and accurate trait analyses with minimal sample preparation as compared to traditional laboratory wet chemistry methods (Alamu et al., 2020; Gaby et al., 2021).

These advances in phenomics offer the potential of transforming cassava breeding into a data‐rich, evidence‐driven process, an important precursor for making genetic gains (Cobb et al., 2019). According to the genetic gain equation (Eberhart, 1970; Lush, 1937), breeding programs can make genetic gains if they are able to either increase their selection intensity (*i*), or selection accuracy (*r*), or reduce on their cycle time (*L*). Therefore, the optimization of NIRS offers the promise of accelerated genetic gains in low HCN cassava breeding.

The only published study on HCN prediction with NIRS in cassava (Sánchez et al., 2014) provided high prospects (i.e., *R* ^2^_C =_ 0.85 and *R* ^2^_p_ = 0.84) using partial least squares regression (PLSR) and quantitative reference data from the enzymatic method (Essers et al., 1993). Although the authors concluded that NIRS could distinguish low and high HCN accessions, the accuracy of predictions in a different population was very low (*R* ^2^_p_ = 0.25), indicating that the model was probably overfitted. Moreover, they did not define what constituted the low or high HCN class. Thus, efforts to predict fresh cassava root HCN content using NIRS have been futile so far.

Machine learning (ML) algorithms use experimental data to develop a model that can classify or predict the behavior of systems (Sampaio et al., 2020). Depending on the dataset, deciding on an algorithm to get the highest performance remains an ad hoc process (Kirasich et al., 2018). Support vector machines (SVMs) might be the most popular ML algorithms given their robustness and good generalizing ability (Awad & Khanna, 2015). Algorithmically, SVMs build optimal separating boundaries between datasets by solving a constrained quadratic optimization problem (Cristianini & Shawe‐Taylor, 2000). NIRS, coupled with SVM, have had diverse applications and have been deployed with high classification accuracies in identification of plant diseases (Mishra et al., 2012), predicting forage quality (Baath et al., 2020), distinguishing transgenic and non‐transgenic Brassica (Sohn et al., 2022), and predicting storage conditions and time after harvest for tomatoes (Emsley et al., 2022).

Logistic regression (LR) is another popular ML algorithm in plant phenomics given its ease of use and interpretability of model parameters (Dreiseitl & Ohno‐Machado, 2002). The quantitative response variable is the log of odds of being classified in the *i*th group of a binary or multi‐class classification (Hastie et al., 2009). LR has been successfully deployed in the identification of citrus greening (Mishra et al., 2012) and geographic origin discrimination of millet (Kabir et al., 2021). Partial least squares discriminant analysis (PLS‐DA) is a linear classification tool that builds models based on PLSR algorithm. The algorithm searches for latent variables with maximum covariance to represent the relevant sources of data variability with linear combinations of the original variables. Thus, PLS‐DA models can classify samples based on class probabilities or by calculating class thresholds based on Bayes theorem (Ballabio & Consonni, 2013). PLS‐DA has been successfully used in determining the floral origin of honey (Akbari et al., 2020), identification of rice flour types (Sampaio et al., 2020), and classification of fruits (Cunha Junior et al., 2015).

Despite their immense potential, ML algorithms have not been explored for categorizing HCN content in fresh cassava roots. Developing and adapting enhanced NIRS prediction models for HCN would enable breeding programs to screen large segregating populations right from the early stages, a process not done before. This study, therefore, sought to assess the predictive accuracy of NIRS for fresh root HCN content using ML algorithms, that is, LR, SVM, and PLS‐DA.

**Core Ideas**

- NIRS combined with machine learning algorithms can discriminate low and high HCN cassava accessions with high classification accuracy.
- The wave lengths 961, 1165, 1403–1505, 1913–1981, and 2491 nm are influential in discrimination of low and high HCN accessions.
- Spectral pre‐treatments can increase classification accuracy of machine learning algorithms such as logistic regression and PLS‐DA using the full spectrum range (400–2500 nm).
- NIRS as an accurate, high throughput tool is key for breeding programs to achieve genetic gains in low HCN cassava breeding.

## 2 MATERIALS AND METHODS

### 2.1 Test accessions

A total of 1543 accessions, consisting of pre‐breeding white, cream, and yellow root‐fleshed populations, were utilized in this study. These populations were developed at the National Crops Resources Research Institute (NaCRRI) in Uganda. Separate field trials were conducted at two locations: Namulonge in the central region, where 1197 clones were evaluated and at Ngetta in the northern region, where 346 clones were evaluated. All trials followed an augmented design and were planted during the 2020/2021 season. Each plot consisted of 10 plants, with a spacing of 1 m between plants and 1 m between adjacent rows. Namulonge is characterized by sandy‐loam soils and has a mean annual temperature of 24°C, while Ngetta is characterized by sandy‐loam soils with a mean annual temperature of 26°C. Throughout the trials, efforts were made to maintain weed‐free conditions through regular weeding. No fertilizer was applied during the study.

### 2.2 NIRS spectra collection

The NIRS spectra were collected following the protocol by Kanaabi et al. (2023). At 12 months after planting, the middle five plants of each plot were harvested and their roots were pooled together. Three uniformly sized, non‐necrotic roots were selected per plot and taken to the laboratory from this pool. The roots were washed under running water to remove debris and dried on the surface with a kitchen towel. To prepare the samples, a 5‐cm long cross‐section from the center of each root was cut, peeled with a kitchen knife, and grated using a kitchen grater (Tablecraft SG205BH 9″). NIRS spectra were collected from the grated sample using a filled small sample cup of the FOSS NIRS TM (Model DS2500, Serial No. 91793020). The spectra were recorded from 400 to 2500 nm at 8.5 nm intervals and saved as the average of 32 scans per root. Because only non‐necrotic roots were used, spectra were collected from only 585 diverse accessions (413 in Namulonge and 172 in Ngetta). Two spectra were taken per root, these were averaged and saved as one spectrum from each of the three roots for each accession. However, spectra were collected from only one or two suitable roots for some accessions. In total, 1564 spectra were collected and utilized in downstream analyses.

### 2.3 Reference data collection and spectra pretreatment

Reference data for HCN was collected using the picrate paper method (Fukuda et al., 2010) as described by Kanaabi et al. (2023). The picrate method uses a 1–9 visual scale in which 1 and 9 represent low and high HCN content extremes, respectively. Samples scoring <6 typically contain <60 ppm of HCN, while those scoring ≥6 typically contain ≥60 ppm HCN (Hershey, 2020). The sample used was drawn from the same sample scanned by NIRS. Therefore, we collected the corresponding reference HCN data. Reference data were collected in duplicate for each root sample. Overall, 1564 average HCN data points were collected. The spectra were subjected to different spectral pretreatments including standard normal variate (SNV) (Barnes et al., 1989), Savitzky–Golay (SG) smoothing (Chen et al., 2013), Savitzky–Golay with standard normal variate (SG.SNV), standard normal variate with first derivative (SNV.d1), standard normal variate with second derivative (SNV.d2), Savitzky–Golay with first derivative, window 5 (SG.d1,w5), and Savitzky–Golay with second derivative, window 5 (SG.d2,w5).

### 2.4 Binary classification of HCN

Based on average HCN scores, samples were categorized as low HCN (i.e., average score 1.0–5.9) and high HCN (i.e., average score 6.0–9.0). Principle component analysis (PCA) was performed in R statistical package (R Core Team, 2023) using the *prcomp* function, and a PCA of individuals was plotted on the untreated spectra using the GGally package to visualize how individuals grouped based on the NIRS spectral profile. Furthermore, the PCA loadings were plotted against wavelength to identify potentially influential wavelengths. Therefore, downstream analyses were separately conducted both on selected wavelengths and on the full spectral range (400–2500 nm).

The NIRS spectra were further used to test ML algorithms—LR, SVM, and PLS‐DA—for their ability to distinguish between low and high HCN accessions using XLSTAT software. Every algorithm was tested on the spectra obtained from the 1564 root samples for the different spectra pretreatments. Data were randomly divided into a training set (70%) and a validation set (30%). Thus, the training set had 1094 samples, while the validation set had 470 samples. For LR and SVM models, 10‐fold cross validation was performed. For PLS‐DA, Jackknife cross validation was performed with five groups. For LR, binary classification was performed using a Logit model in Newton Raphson algorithm with the model parameters as follows: convergence = 0.000001, number of iterations = 10, confidence interval = 95%, tolerance = 0.001, and cut point = 0.5. SVM binary classification was performed on rescaled spectra using a liner kernel, with the C parameter set to 100 and tolerance set to 0.001.

### 2.5 ML model performance metrics

High‐HCN class was used as positive, while low‐HCN class was used as control group (negative) in evaluating ML models. These were evaluated according to accuracy, precision, sensitivity, and specificity. Accuracy denotes the ratio of number of correct predictions to total number of input samples, while precision refers to ratio of true positives to predicted positives (Sokolova et al., 2006). Sensitivity denotes ratio of true positives to total number of samples classified as positives, while specificity denotes true negatives as a ratio of all samples classified as negatives. The *F*‐score is a harmonic mean of sensitivity and precision. It measures a model's accuracy (Sokolova et al., 2006).

$$\mathrm{Accuracy}\;=\frac{\mathrm{True}\;\mathrm{positives}\;\left(\mathrm{TP}\right)+\mathrm{True}\;\mathrm{Negatives}\;\left(\mathrm{TN}\right)}{\mathrm{True}\;\mathrm{positives}\;\left(\mathrm{TP}\right)+\mathrm{True}\;\mathrm{Negatives}\;\left(\mathrm{TN}\right)+\mathrm{False}\;\mathrm{Positives}\;\left(\mathrm{FP}\right)+\mathrm{False}\;\mathrm{Negatives}\;\left(\mathrm{FN}\right)}.\;$$

Average accuracy was computed as the mean of the accuracy of ten model runs.

Classificationerrorrate=1−Accuracy,

$$\mathrm{Precision}\;=\frac{\mathrm{TP}}{\mathrm{TP}+\mathrm{FP}}\;,$$

$$\mathrm{Sensitivity}\;=\frac{\;\mathrm{TP}}{\mathrm{TP}+\mathrm{FN}}\;,$$

$$\mathrm{Specificity}\;=\frac{\mathrm{TN}}{\mathrm{TN}+\mathrm{FP}}\;.$$

Analysis of variance (ANOVA) was conducted to test for the significance of observed differences in performance of ML algorithms and spectra pretreatments at 5% alpha level. Means were separated using Tukey's honest significant difference test implemented in the Agricolae package in R.

## 3 RESULTS AND DISCUSSION

The overall objectives of this study were to (i) access the potential of NIRS for discrimination of low and high HCN cassava accessions using the ML algorithms LR, SVM, and partials least squares discriminant analysis and (ii) access the effect of spectra pretreatments on performance of the ML algorithms. This was done by collecting NIRS spectra from 1164 fresh cassava root samples and corresponding reference laboratory data. These datasets were then used to assess the potential ML algorithms for binary classification of samples.

### 3.1 Spectra pattern of fresh grated cassava roots

**FIGURE 1:**
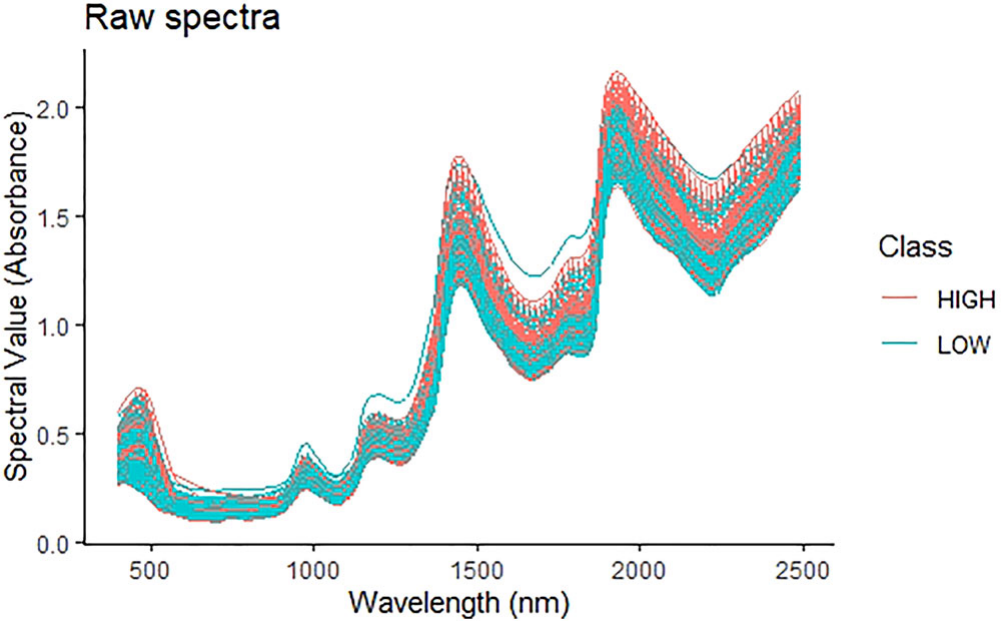
Near‐infrared spectroscopy (NIRS) spectra of 1564 fresh cassava root samples drawn from high and low hydrogen cyanide potential (HCN) accessions.

Organic molecules absorb NIR radiation primarily due to overtone and combination bands of O‐H, C‐H, N‐H, and C = O groups. When the spectra were plotted, distinct peaks were observed at approximately 500, 1000, 1200, 1500, 2000, and 2100–2500 nm (Figure 1). The peak at 500 nm in the visible region was attributed to colored compounds in the samples because some of the roots had yellow or cream parenchyma. The peaks around 1500 and 2000 nm are typical of water, as fresh cassava roots contain 60%–75% water (Kajuna et al., 2001). Furthermore, peaks were observed at 1000 nm corresponding to the C‐H third and O‐H second overtone; around 1200 nm corresponding to the second C‐H overtone; between 1300 and 1500 nm corresponding to the first overtone of C‐H combination, first O‐H overtone, and first N‐H overtone; between 1800 and 2000 nm corresponding to C + O + O + H combinations and O‐H combinations; and between 2100 and 2500 nm corresponding to ROH, RNH_2_, CONH_2_(H), CC, CHO, CH_2_, and CH_3_ combinations (Eldin, 2010). A slight pattern of separation between low and high HCN accessions was evident on the spectral plot (Figure 1).

Although a slight pattern of separation of classes is observed in Figure 1, it was impossible to solely use a plot of the NIRS spectral profile to separate the low and high HCN accessions. Therefore, several ML methods, including PCA, SVM, LR, and PLS‐DA, were evaluated for their ability to discriminate between low and high HCN accessions.

### 3.2 Principal component analysis

PCA is an unsupervised ML algorithm that reduces a spectral dataset into a small number of principle components (PCs) that explain most of the variance in the original dataset (Jolliffe, 2002). Each PC is orthogonal to the other and consists of scores and loadings. The scores represent variance in sample direction and are used to identify patterns of similarity between samples, while the loadings represent variance in the wavelength direction (Emsley et al., 2022). Thus, PC 1 (74.4%) and PC 2 (17.4%) cumulatively explained 91.8% of the variation in NIRS spectra. Up to 99% of the total variation in spectra was explained by the first six PCs; with PC 3 (4.2%), PC 4 (2%), PC 5 (0.8%), and PC 6 (0.4%) cumulatively explaining 7.4% of the variation.

**FIGURE 2:**
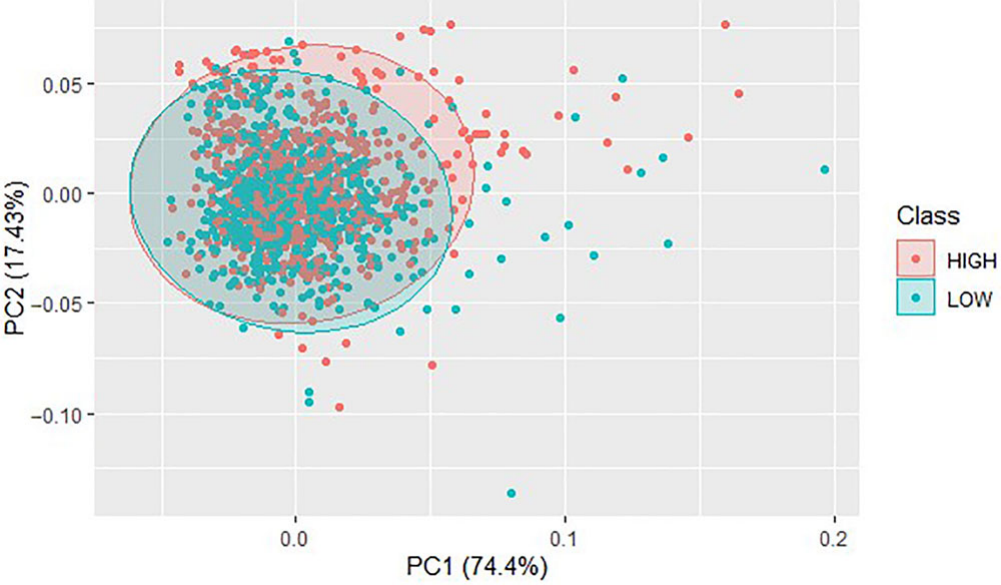
Principal component analysis (PCA) score biplot showing clustering of high and low hydrogen cyanide potential (HCN) accessions using raw spectra of 1564 samples. The axes are the first principal component (PC1) and the second principal component (PC2).

However, when we plotted the PCA score plot, there was only a slight separation pattern between the high and low HCN classes (Figure 2). This separation pattern, albeit slight, suggests that there could be chemical differences between high and low HCN accessions (Kabir et al., 2021; Sampaio et al., 2020).

### 3.3 Variance in wavelength direction

**FIGURE 3:**
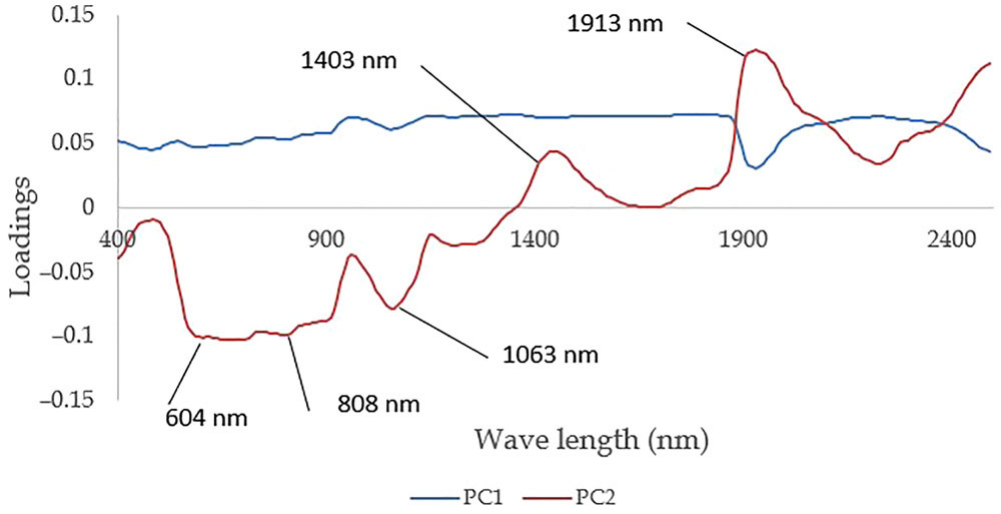
A plot of principal component analysis (PCA) loadings of the first principal component (PC1) and second principal component (PC2) against wavelength showing potentially influential wavelengths in discriminating high and low hydrogen cyanide potential (HCN) samples.

A plot of the PCA loadings against wavelength shows regions 961, 1165, 1403–1505, 1913–1981, and 2491 nm as having the highest values of PCA loadings for PC 2 (Figure 3). These regions could be responsible for the variation between low and high HCN samples. Thus, initial assessments of performance of ML algorithms were performed using these wavelengths.

It was evident from the PCA biplot (Figure 3) that PCA could not decisively separate low and high HCN groups using NIRS spectra. The inability of PCA to decisively distinguish samples based on their NIR spectral pattern has been widely reported (Currò et al., 2021; Kabir et al., 2021; Qiu et al., 2019; Sampaio et al., 2020; Sohn et al., 2022). Given this setback with PCA, all the studies developed supervised ML classification models that effectively discriminated their samples. Thus, in this study, more robust ML algorithms SVM, LR and PLS‐DA were examined for their potential discriminate low and high HCN accessions. Both raw and pretreated spectra were used on the selected wavelengths (961, 1165, 1403–1505, 1913–1981, and 2491 nm) as well as on the full spectral wavelength (400–2500 nm).

### 3.4 Performance matrices of ML algorithms for binary classification of fresh cassava root HCN content based on selected wavelengths

241 Classification models differed significantly (*p* < 0.001) in their classification accuracy. The LR algorithm was the most accurate (100%), closely followed by PLS‐DA (99.6%; Table 1). However, SVM performed significantly differently from the two algorithms with only 64.6% accuracy. For all evaluated model performance parameters, LR scored 100%, while PLS‐DA scored 99%. The performance of the SVM algorithm was not comparable to LR and PLS‐DA for all the evaluated model performance parameters (Table 1). The spectral pretreatments had no significant effect (*p* = 0.98) on ML algorithm prediction accuracy. The LR and PLS‐DA models had *F*‐scores of 1 and 0.99, respectively, indicating a high degree of accuracy of these models, while the SVM had an *F*‐score of 0.56, which indicates that the model was only moderately accurate. Sensitivity is a measure of high HCN samples that were predicted as high HCN. The LR (1) and PLS‐DA (0.99) models had high sensitivity, therefore could correctly classify as high HCN samples. On the other hand, with a sensitivity of only 0.51, the SVM model could only correctly classify high HCN samples 51% of the times. Specificity on the other hand provides a measure of low HCN samples that were correctly identified as low HCN (Table 1). The LR (1) and PLS‐DA (0.99) models had high specificity scores. The SVM algorithm (0.77) had a moderate ability to correctly classify low HCN samples. Precision is a measure of the proportion of predicted high HCN samples that were actually high in HCN, an indicator of the model's ability to avoid false positives. The LR (1) and PLS‐DA (0.99) had high precision, while SVM (0.68) had a low precision (Table 1).

**TABLE 1:** Performance matrices of machine learning algorithms for binary classification of fresh cassava root hydrogen cyanide potential (HCN) content based on selected wavelengths—961, 1165, 1403, 1403–1505, 1913–1981, and 2491 nm.

	Classification accuracy (%)				*F*‐**score**				Sensitivity				Specificity				Precision			
ML algorithm	Mean	SD (%)	Min (%)	Max (%)	Mean	SD	Min	Max	Mean	SD	Min	Max	Mean	SD	Min	Max	Mean	SD	Min	Max
LR	100a	0	100	100	1.00a	0.00	1.00	1.00	1.00a	0.00	1.00	1.00	1.00a	0.00	1.00	1.00	1.00a	0.00	1.00	1.00
PLS‐DA	99.6a	1.2	93	100	0.99a	0.02	0.9	1.00	0.99a	0.01	0.95	1.00	0.99a	0.02	0.90	1.00	0.99a	0.02	0.90	1.00
SVM	64.6b	3.6	54	73	0.56b	0.14	0.15	0.75	0.51b	0.18	0.08	0.80	0.77b	0.12	0.47	0.98	0.68b	0.06	0.57	0.82

*Note*: Means followed by the same letter are not significantly different at *ă* = 0.05.

Abbreviations: LR, logistic regression; Max, maximum; Min, minimum; ML, machine learning; PLS‐DA, partial least squares discriminant analysis; SD, standard deviation; SVM, support vector machine.

### 3.5 Prediction accuracy of ML algorithms using the full spectral range (400–2500 nm)

Results indicated that there were moderate to high prediction accuracies for the different model‐spectra pretreatment combinations when all spectra were used. There were significant differences (*p* < 0.001) in the prediction accuracies among the models. Similarly, we found that the spectral pretreatments had significant differences (*p* < 0.001) that were associated with significant differences in the prediction accuracies. We also observed that the accuracy of the models depended on the spectra pretreatment administered (*p* < 0.001). Overall, PLS‐DA had the highest prediction accuracy (90.03%), followed by SVM (74.82%) and LR (74.12%). SVM and LR had similar mean accuracies (Table 2). SNV.d2 (84.53%) had the highest mean accuracy for spectra pretreatments. SG (81.98%) and SG.d2,w5 (80.80%) had similar accuracy with the raw spectra (81.98%). The other pretreatments had mean accuracies that were significantly lower than those of the raw spectra (Table 2).

**TABLE 2:** Mean overall prediction accuracies of machine learning models and spectra pretreatments.

Machine learning models	Classification accuracy (%)	SD (%)	Min (%)	Max (%)
**Model**				
PLS‐DA	90.03a	11.99	62.98	100
SVM	74.82b	3.17	67.87	82.34
LR	74.12b	7.22	43.83	79.36
**Spectra pretreatments**				
Raw	81.98ab	12.67	67.87	100
SG	81.98ab	12.72	68.51	100
SG.d1,w5	80.32b	9.58	71.06	99.15
SG.d2,w5	80.80ab	7.62	68.51	94.04
SG.SNV	78.52bc	7.92	62.98	93.62
SNV	75.07cd	15.26	43.83	100
SNV.d1	74.06d	5.16	64.26	82.34
SNV.d2	84.53a	10.91	73.83	100

*Note*: Means followed by the same letter are not significantly different at *ă* = 0.05.

Abbreviations: LR, logistic regression; Max, maximum classification accuracy; Min, minimum classification accuracy; PLS‐DA, partial least squares discriminant analysis; Raw, no spectra pretreatment; SD, standard deviation; SG, Savitzky–Golay; SG.d1,w5, Savitzky–Golay with first derivative, window 5; SG.d2,w5, Savitzky–Golay with second derivative, window 5; SG.SNV, Savitzky–Golay with standard normal variate; SNV, standard normal variate; SNV.d1, standard normal variate with first derivative; SNV.d2, standard normal variate with second derivative, SVM, support vector machine.

When influential wavelengths were identified and used for binary classification, LR (100%) and PLS‐DA (99%) yielded highly accurate models. The accuracy of the classification model for SVM (64.6%) was moderate but less than that of the full spectral range (75%). The overall accuracy of PLS‐DA improved from 90% to 99%, while that of LR improved from 74% to 100% (Tables 1 and 2). Although earlier authors working on cassava frog skin disease (Freitas et al., 2020) reported no improvement in the classification accuracy of six classification models using selected influential wavelengths; in this study, the prediction accuracies of PLS‐DA and LR algorithms were greatly improved when influential wavelengths were selected (Tables 1 and 2), thus underpinning the importance of identifying and using key wavelengths in developing ML classification models using NIRS spectra. Elsewhere, Sampaio et al. (2020) reported the wavelengths 5478, 4215, and 7372–7324 cm^−1^ to be influential in discrimination of rice flour types, while Migacz et al. (2022) reported that the region 2000–2500 nm was essential in differentiation of *Eucalyptus* species. Reducing the number of wavelengths used in classification model development using large multi‐dimensional datasets like NIRS has the additional advantage of reducing the computational power required for complex algorithms like SVM, thus making it appealing to even resource limited breeding programs.

### 3.6 Effect of spectra pretreatments on classification accuracy of ML models using the full spectral range (400–2500 nm)

Since the interaction of spectra pretreatment with the model was highly significant (*p* < 0.001), we examined the effect of the different spectra pretreatments on the accuracy of the models. We observed moderate to high prediction accuracies for the different model‐spectra pretreatment combinations. PLS‐DA was the most efficient model for discriminating low and high HCN accessions using raw and pretreated spectra. Using raw spectra, average prediction accuracy for PLS‐DA was 99.3%. Using treated spectra, average accuracy of PLS‐DA was 99.4% for SG; 91.2% for SG.d1,w5; 87.1% for SG.d2,w5; 85.1% for SG.SNV; 67.8% for SNV.d1; and 99.6% for SNV.d2 (Table 3). Raw spectra and the pretreatments SNV.d2 and SG produced stable models with high accuracy, while SG with first derivative and SG.SNV produced models with high variability (Table 3).

**TABLE 3:** Mean classification accuracy of machine learning models with different spectral pretreatments for the validation set.

Model	Pretreatment	Mean classification accuracy (%)	Min (%)	Max (%)	SD (%)
PLS‐DA	Raw	99.28ab	98.3	100	0.59
	SG	99.43a	98.51	100	0.47
	SG.d1,w5	89.68bc	71.06	99.15	11.99
	SG.d2,w5	88.25c	68.51	94.04	9.44
	SG.SNV	85.15c	62.98	93.62	10.95
	SNV	91.02abc	84.04	100	5.25
	SNV.d1	67.81d	64.26	72.55	2.68
	SNV.d2	99.60a	99.15	100	0.27
LR	Raw	75.70a	74.04	77.87	1.29
	SG	74.64a	70.43	78.09	2.26
	SG.d1,w5	75.06a	72.98	79.36	2.11
	SG.d2,w5	77.32a	74.89	79.15	1.34
	SG.SNV	76.45a	73.40	78.94	1.58
	SNV	60.83b	43.83	79.36	14.28
	SNV.d1	75.72a	72.34	78.3	2.07
	SNV.d2	77.32a	73.83	79.15	1.75
SVM	Raw	70.96d	67.87	74.68	2.08
	SG	71.85cd	68.51	74.68	2.16
	SG.d1,w5	76.21ab	74.89	78.09	1.15
	SG.d2,w5	76.83a	74.04	79.79	1.88
	SG.SNV	73.95bc	71.06	76.14	1.75
	SNV	73.37 cd	69.26	78.15	2.79
	SNV.d1	78.64a	74.68	82.34	2.09
	SNV.d2	76.77a	75.32	79.78	1.5

*Note*: Means followed by the same letter are not significantly different at *ă* = 0.05.

Abbreviations: LR, logistic regression; Max, maximum classification accuracy; Min, minimum classification accuracy; PLS‐DA, partial least squares discriminant analysis; Raw, no spectra pretreatment; SD, standard deviation; SG, Savitzky–Golay; SG.d1,w5, Savitzky–Golay with first derivative, window 5; SG.d2,w5, Savitzky–Golay with second derivative, window 5; SG.SNV, Savitzky–Golay with standard normal variate; SNV, standard normal variate; SNV.d1, standard normal variate with first derivative; SNV.d2, standard normal variate with second derivative, SVM, support vector machine.

The prediction accuracies of LR and SVM were moderate and comparable for both raw and pretreated spectra. For raw spectra, prediction accuracies were 71% for SVM and 75.7% for LR. For SG, accuracy was 74.6% (LR) and 72% (SVM), for SG.d1,w5, accuracy was 75.1% for LR and 76.2% for SVM, while for SG.d2,w5, accuracy was 77.3% for LR and 76.8% for SVM. For SG.SNV, accuracy was 76.4% for LR and 74% for SNV. With SNV, accuracy was 60.8% for LR and 73.4% for SVM, while for SNV.d1, accuracy was 75.7% for LR and 78.6% with SVM. With SNV and second derivative, accuracy was 77.2% for LR and 76.7% with SVM (Table 3).

Overall, using the full spectral range, PLS‐DA combined with SNV and second derivative (99.60%) and PLS‐DA combined with SG (99.43%) produced the most accurate models. PLS‐DA combined with raw spectra (99.28%) outperformed all the remaining model‐spectra pretreatment combinations (Table 3).

Despite the overall superior performance of PLS‐DA to LR and SVM using the full spectrum, the two models—LR (75.72%) and SVM (78.64%)—outperformed PLS‐DA (67.81%) when SNV.d1 spectral pretreatment was applied, yet PLS‐DA achieved 99.38% accuracy with raw spectra (Table 3). Although spectra pretreatments correct for systemic noise and could highlight differences between samples (Freitas et al., 2020; Sampaio et al., 2020; Sohn et al., 2022), care should be taken to identify the best pretreatment for a given spectral dataset, else the pretreatments could disrupt the pattern in the spectra (Nkouaya Mbanjo et al., 2022).

### 3.7 Classification model performance parameters using the full spectral wavelength (400–2500 nm)

The models were further evaluated for the different spectra pretreatments' precision, sensitivity, specificity, and *F*‐score. Overall, PLS‐DA was superior to LR and SVM, achieving values of 0.99 for all four parameters with raw spectra, SNV.d2, SG, and SG.SNV pretreatments (Table 4). These values were significantly higher than those of other pretreatments (Table 4). For SVM, precision ranged from 0.75 with raw spectra to 0.79 with no significant difference between raw and pretreated spectra. Similarly for LR, precision ranged 0.59 (SNV) to 0.88 (SG.d2,w5). Only SG.d2.w5 was significantly different from raw spectra and other pretreatments. With SVM, sensitivity values ranged from 0.63 (SG) to 0.77 with SNV.d1, while for LR it ranged from 0.63 (SNV) to 0.89 (SG.d2,w5). The *F*‐scores ranged 0.46–0.78 for SVM algorithm, while they ranged 0.6–0.88 with LR (Table 4).

**TABLE 4:** Classification model performance parameters.

Model	Pretreatment	Precision	Sensitivity	Specificity	*F*‐**score**
PLS‐DA	Raw	0.99a	0.99a	0.99a	0.99a
	SNV	0.86bc	0.88ab	0.82bc	0.87bc
	SNV.d1	0.87bc	0.89ab	0.85bc	0.88bc
	SNV.d2	0.99a	0.99a	0.99a	0.99a
	SG	0.99a	0.99a	0.99a	0.99a
	SG.d1,w5	0.91ab	0.86b	0.93ab	0.92ab
	SG.d2,w5	0.76c	0.80b	0.75c	0.78c
	SG.SNV	0.99a	0.99a	0.99a	0.99a
SVM	Raw	0.75a	0.64b	0.77a	0.67c
	SNV	0.77a	0.64b	0.82a	0.46d
	SNV.d1	0.79a	0.77a	0.80a	0.78a
	SNV.d2	0.78a	0.73ab	0.80a	0.75a
	SG	0.75a	0.63b	0.80a	0.67c
	SG.d1,w5	0.77a	0.72ab	0.80a	0.74ab
	SG.d2,w5	0.79a	0.75ab	0.79a	0.76a
	SG.SNV	0.76a	0.65ab	0.80a	0.70bc
LR	Raw	0.75b	0.76b	0.74b	0.76b
	SNV	0.59c	0.63c	0.59c	0.60c
	SNV.d1	0.75b	0.78b	0.73b	0.76b
	SNV.d2	0.76b	0.80ab	0.74b	0.78b
	SG	0.75b	0.77b	0.72b	0.76b
	SG.d1,w5	0.76b	0.79b	0.71b	0.76b
	SG.d2,w5	0.88a	0.89a	0.87a	0.88a
	SG.SNV	0.75b	0.77b	0.76b	0.76b

*Note*: Means followed by the same letter are not significantly different at *ă* = 0.05.

Abbreviations: LR, logistic regression; PLS‐DA, partial least squares discriminant analysis; Raw, no spectra pretreatment; SG, Savitzky–Golay; SG.d1,w5, Savitzky–Golay with first derivative, window 5; SG.d2,w5, Savitzky–Golay with second derivative, window 5; SG.SNV, Savitzky–Golay with standard normal variate; SNV, standard normal variate; SNV.d1, standard normal variate with first derivative; SNV.d2, standard normal variate with second derivative, SVM, support vector machine.

The accuracy reported for the LR algorithm using selected wavelengths in this study (100%) is comparable to that reported by Kabir et al. (2021), who reported a classification accuracy of 98.8% when they attempted to discriminate the geographic origin of millet. Similarly, the classification accuracy based on the full spectrum (74%) is comparable to 81% classification accuracy that was reported by Mishra et al. (2012) when they used LR to discriminate healthy plants from those infected by citrus greening disease. The high classification accuracies reported for PLS‐DA (99%) in this study are consistent with reports from earlier classification studies for different applications. Akbari et al. (2020) used PLS‐DA to determine the floral origin of honey and achieved 100% classification accuracy. Similarly, Lapcharoensuk et al. (2023) used PLS‐DA for detection of pesticide residues in bok choi vegetable, achieving a classification accuracy of 99%–100% with different spectral pretreatments, while Qiu et al. (2019) achieved accuracy of 99.19% when they attempted cultivar classification of single corn seed.

### 3.8 Usefulness of binary classification of low and high HCN accessions in routine cassava breeding

The HCN content of cassava profoundly impacts the adoption and use of cassava varieties. Thus, farmers can reliably classify their cassava into “bitter” and “cool” varieties (Mkumbira et al., 2003). Although most communities in subsistence agricultural systems have preference for non‐bitter varieties (Iragaba et al., 2021), there are isolated communities that prefer bitter varieties because they perceive them to be less susceptible to pest damage and human theft (Mkumbira et al., 2003; Nakabonge, 2018). However, given resource limitations, breeding programs in such regions concentrate on breeding for low HCN accessions given the dangers associated with consumption of high HCN cassava (Alitubeera et al., 2019; Teles, 2002), but also given the fact that sweet varieties characterized by low HCN are a preferred attribute in communities whose staple is boiled cassava. In more industrialized economies like Brazil, some regions typically cultivate bitter varieties for industrial starch extraction, while others cultivate non‐bitter varieties for food (Ogbonna et al., 2021). This ensures that the delicate balance between cassava production for food and production for industry is maintained. Thus, we must have fast and accurate methods to discriminate low and high HCN accessions.

In this study, we have demonstrated that NIRS, combined with ML algorithms, can effectively separate low and high HCN accessions, achieving classification accuracy of up to 99% with PLS‐DA and 100% with LR. Using NIRS, up to 60 duplicate samples can be analyzed in a day. Compared to the reference method (Fukuda et al., 2010), results on a similar sample number can only be obtained after 12–15 h. With the advantage of increase throughput and accuracy (Alamu et al., 2020), NIRS could help breeding programs to make genetic gains in breeding for low HCN cassava varieties by allowing them to screen more accessions at reduced cost and increased accuracy, thus increasing their selection intensity and accuracy. Moreover, NIRS as a high throughput phenotyping tool fits well into current efforts by major cassava breeding programs collaborating under the Nextgen cassava breeding project (www.nextgencassava.org) to modernize their operations through genomic selection. Ikeogu et al. (2019) demonstrated that NIRS predicted phenotypes could be used to build genomic prediction models.

Given the moderate to high heritability reported for HCN (Ogbonna et al., 2021; Torres et al., 2021), lowering population HCN levels through breeding is attainable by selection of low HCN parents and progeny screening from early stages (Hershey, 2020). A high throughput method that discriminates low from high HCN accessions will facilitate the selection of low HCN accessions in diverse early‐stage seedling and/or clonal trials. This ensures two principal outcomes: (1) early identification of low HCN parents to be used for breeding and (2) advancement of only low HCN accessions in the cassava for boiled food breeding pipeline. This minimizes resource wastage as high HCN accessions would have to be discarded anyway at later stages of the breeding pipeline of cassava for boiled food.

If high HCN accessions are identified in early breeding stages, they can as well be considered for other market segments like brewing. At later stages of breeding, starting with the uniform yield trials when accession numbers are small, laboratory reference methods could be deployed to generate quantitative data on exact fresh root HCN content for variety description in support of the variety release process.

The high throughput method based on NIRS offers choice in addition to being very useful in marker‐assisted selection for low HCN cassava. KASP markers for HCN (low HCN/high HCN classification) were developed (Ogbonna et al., 2021) but these would have to first be validated with individual breeding program populations, a process that involves collection of vast volumes of phenotypic data.

These results are also useful for public health and safety regulators as NIRS could be readily deployed to access the safety in relation to HCN content categorization of cassava roots sold to consumers in local markets and/or those destined for export. To our knowledge, this is the first attempt to use NIRS combined with ML algorithms for phenotyping of cassava root quality traits. This provides frameworks for its advancement for other important cassava root quality traits, that is, mealiness and fibrousness.

## 4 CONCLUSIONS

Based on datasets generated from this study, three conclusions are apparent. First, the wavelengths 961, 1165, 1403–1505, 1913–1981, and 2491 nm are influential in discriminating low and high HCN cassava accessions. Second, the ML algorithms LR and PLS‐DA can achieve prediction accuracies of 100% and 99%, respectively, when classification models are developed using selected influential wavelengths. Third, PLS‐DA can achieve up to 99% classification accuracy with the full range of spectra wavelength (400–2500 nm) using either raw spectra or raw spectra combined with SNV.d2 spectral pretreatment. NIRS combined with LR or PLS‐DA algorithm could be deployed in routine phenotyping and selection of low HCN cassava accessions.

## AUTHOR CONTRIBUTIONS

**Michael Kanaabi**: Conceptualization; data curation; formal analysis; investigation; methodology; software; visualization; writing—original draft. **Fatumah B. Namakula**: Data curation; formal analysis; software; visualization; writing—review and editing. **Ephraim Nuwamanya**: Conceptualization; investigation; methodology; resources; supervision; validation; writing—review and editing. **Ismail S. Kayondo**: Conceptualization; investigation; methodology; resources; writing—review and editing. **Settumba Mukasa**: Conceptualization; investigation; methodology; resources; supervision; validation; writing—review and editing. **Nicholas Muhumuza**: Data curation; investigation; methodology. **Enoch Wembabazi**: Investigation; writing—review and editing. **Paula Iragaba**: Conceptualization; methodology; writing—review and editing. **Leah Nandudu**: Conceptualization; methodology; writing—review and editing. **Ann Ritah Nanyonjo**: Investigation; methodology; writing—review and editing. **Julius Baguma**: Investigation; methodology; writing—review and editing. **Esuma Williams**: Writing—review and editing. **Alfred Ozimati**: Writing—review and editing. **Titus Alicai**: Conceptualization; methodology; supervision. **Angele Ibanda**: Conceptualization; funding acquisition. **Robert S. Kawuki**: Conceptualization; funding acquisition; investigation; methodology; project administration; resources; supervision; validation; writing—review and editing.

## CONFLICT OF INTEREST STATEMENT

The authors declare no conflicts of interest.

## Data Availability

Data used in this manuscript are available in an open access online data repository Cassavabase at: https://cassavabase.org/breeders/trials/.
